# Evaluating the Efficacy and Safety of the LASIK Nonlinear Aspheric Micro-Monovision Surgery (Laser Blended Vision Surgery) in Correcting Presbyopia Among the Saudi Population

**DOI:** 10.3390/healthcare14091232

**Published:** 2026-05-03

**Authors:** Mohammed M. Althomali, Mohamed G. Eissa, Faisal N. Almushaweh, Ahmed A. Alharbi, Muteb K. Alanazi, Waleed S. AlTuwairqi

**Affiliations:** 1 Optometry Department, College of Applied Medical Sciences, King Saud University, Riyadh 11362, Saudi Arabia; 2 Department of Ophthalmology, Kasr Al Ainy Hospital, Faculty of Medicine, Cairo University, Cario 11431, Egypt; 3 Elite Medical and Surgical Center, Riyadh 11527, Saudi Arabia

**Keywords:** PRESBYOND, Laser Blended Vision, LBV, presbyopia

## Abstract

Background/Objectives: To evaluate the monocular and binocular visual outcomes for patients who underwent PRESBYOND Laser Blended Vision (LBV) surgery. Methods: A total of 46 patients participated in this study (mean age 47.5 yrs ± 4.7) with various refractive errors. Patients were treated using the MEL 90 excimer laser to correct their refractive errors. The patients were asked to attend follow-up sessions at intervals of 1, 3, and 6 months. A self-developed binary (Yes/No) satisfaction survey was then conducted at the end of the 6-month visit. Results: The mean preoperative spherical equivalent for the distance eyes was −0.42 ± 1.53, and for the near eyes was −0.4 ± 1.76. The mean logMAR for the uncorrected distance visual acuity after treatment and follow-up at one month, three months, and six months was 0.016, 0.037, and 0.028 (20/20 to 20/22 range), respectively. After treatment, 81% of patients achieved 20/22.5 vision or better, and 94% achieved 20/25 vision or better. For near vision, 100% of patients reached J2 print. After treatment, 97% of patients achieved 20/25 or better at distance and J2 or better at near. Patient satisfaction was 92%, with only 8% of patients reporting dissatisfaction. Conclusions: This protocol has demonstrated stable, well-tolerated, effective, and acceptable results in patients with presbyopia. The safety and efficacy of the procedure were demonstrated, with strong outcomes.

## 1. Introduction

Presbyopia is a physiological age-related condition that affects older adults after the age of 40 due to the gradual decline of accommodation power that is being exerted within the crystalline lens. It is estimated that more than 1.8 billion people are affected by presbyopia globally [1]. Different approaches have been proposed to address this loss of accommodation, including spectacle use, contact lenses, pharmacological treatments, or surgical techniques involving the cornea or the lens [2,3].

Corneal surgical techniques for presbyopia correction have evolved from conventional monovision approaches toward procedures designed to improve the range of functional vision while preserving visual quality. Monovision LASIK has been widely used because of its relative simplicity and effectiveness in reducing spectacle dependence; however, it may be associated with reductions in stereopsis and contrast sensitivity [4]. Other corneal approaches have been developed, including presbyopia laser-assisted in situ keratomileusis (presbyLASIK), conductive keratoplasty, corneal inlays, small incision lenticule extraction (SMILE), and corneal shrinking [5,6,7,8]. More recently, PRESBYOND Laser Blended Vision (LBV) (Carl Zeiss Meditec) has been introduced as a corneal refractive approach to extend the depth of focus through a nonlinear aspheric profile combined with a micro-monovision protocol. This approach was shown to be successful across different degrees of ametropia, with high satisfaction among patients and with minimal compromise in contrast sensitivity or stereoacuity [2,3,9,10,11].

Several studies have shown the efficacy and safety of this procedure among different populations and across different varieties of ametropia. However, this has not yet been explored in the Saudi population. Therefore, the aim of this study was to evaluate the efficacy, accuracy, safety, stability, and satisfaction rate among Saudi patients with emmetropia, hyperopia, and myopia after PRESBYOND LBV surgery over a 6-month follow-up period.

## 2. Materials and Methods

### 2.1. Patients

This study was conducted at Elite Hospital in Riyadh, Saudi Arabia. A retrospective analysis was conducted on patients who underwent treatment for presbyopia with myopia, hyperopia, and emmetropia from July 2021 to July 2022. Ethical approval was acquired through the Institutional Review Board (IRB) at the School of Medicine at King Saud University (Ref. No. 24/0007/IRB-A). Informed consent was obtained from all patients to use their clinical data for publication and seminar purposes.

The inclusion criteria were as follows: aged 40 years and above, eligible for Laser in Situ Keratomileusis (LASIK) surgery, and corrected distance visual acuity (CDVA) of 20/25 or better in both eyes. Patients were excluded if they had any ocular or retinal pathologies, corneal thinning, or corneal or lens opacities. Only patients who attended their follow-up sessions 6 months postoperatively were included in the analysis of this study.

The patients’ eyes were classified as emmetropic if the preoperative spherical equivalent refraction values were (−1.00 D ≥ x ≤ +1.0), myopic > −1.00 D, and Hyperopic > +1.00 D.

### 2.2. Preoperative and Postoperative Evaluation

A comprehensive ocular exam was performed for all patients preoperatively and postoperatively during each follow-up visit. This included uncorrected near visual acuity (UNVA) (Jaeger chart, 40 cm) and uncorrected distance visual acuity (UDVA) (Snellen chart, 6 m), corrected near visual acuity (CNVA), and manifest and cycloplegic refraction. Optical coherence tomography (CSO, Firenze, Italy) and corneal tomography (Schwind eye-tech-solutions, Kleinostheim, Germany) data were collected after using a cycloplegic and mydriatic agent (1% tropicamide). The tomography and keratometry data were also gathered using the Schwind Sirius (Schwind eye-tech-solutions, Kleinostheim, Germany). Patient satisfaction was based on a Yes–No question, with reasons for dissatisfaction, if any.

A bandage contact lens was applied postoperatively, and a broad-spectrum prophylaxis was prescribed for all patients postoperatively. Patients were seen for follow-up at one day, one week, one month, three months, and six months.

### 2.3. Ocular Dominance Test

The dominant eye was determined using four methods: the hole test, pointing, and identifying which eye would be used in the case of using a camera or shooting a rifle [12,13]. In the hole test, the dominant eye was determined by asking the patient to align a distance target binocularly through a hole in a plain sheet. The eyes were then covered alternatively. The eye in which the target was seen was deemed the dominant eye. In pointing, the patient was asked to point at a spot of light 6 m away. The eyes were then covered alternatively, and the eye with the smallest separation between the finger and the light source was considered the dominant eye. The dominant eye was determined if the patient had chosen the same eye for three out of the four tests. When the output of these tests was inconclusive, the patient was asked to use each eye alternatively, and the eye that felt more natural to look at was chosen as the dominant eye.

### 2.4. Micro-Monovision Assessment

Preoperatively, each patient’s tolerance to anisometropia was evaluated to determine the degree of acceptable minimal cross-blurring. The dominant eye was corrected fully for distance, and a +1.50 D lens was placed before the non-dominant eye. Patients viewed both distance and near charts while reporting any perception of blur or visual discomfort. If the patient reported no discomfort, they were asked to read the smallest legible print on both near and distance charts. The examiner then occluded the dominant eye and asked whether the patient had noticed blur in the non-dominant eye at distance. If a negative response is reported, the patient is tolerant to 1.50 D. If cross-blurring was noted, then a reduction of 0.25 D to the anisometropia was applied until the patient reports no cross-blurring. A minimum tolerance of 0.50 D was accepted. In the case that the patient reported no or minimal crossblur with +1.50 D and requires more add, then the anisometropia can also be extended to +1.75 D or +2.00 until the required degree of near vision is attained.

### 2.5. PRESBYOND Treatment

Patients underwent LASIK treatment using the MEL 90 Excimer laser and VisuMax Femtolaser (Carl Zeiss, Meditec AG, Jena, Germany). The aspheric ablation profiles implemented were obtained from the CRS-Master software (Carl Zeiss, Meditec AG, Jena, Germany). The flap thickness was 100 μm with a diameter of 9 mm, while the ablation depth was dependent on the patient’s refractive error. The optical zone ranged from 6 mm to 6.5 mm. All surgical procedures were performed by (WT).

### 2.6. Statistical Analysis

The refractive surgical outcome for this study was derived and calculated following the standard graphs construct defined by Waring and colleagues [14]. Data normality was checked using the Kolmogorov–Smirnov test. For data following a normal distribution, paired *t*-tests were used to compare pre- and postoperative values; otherwise, the Wilcoxon signed-rank test was applied. In addition, 95% confidence intervals (95% CIs) were calculated for key visual outcomes. Comparisons between dominant and non-dominant eyes were performed using paired analyses at the patient level. Microsoft Excel (Microsoft Corporation, Redmond, WA, USA) and SPSS version 28 (SPSS Institute Inc., Chicago, IL, USA) were used for data entry and analysis.

## 3. Results

**Figure 1:**
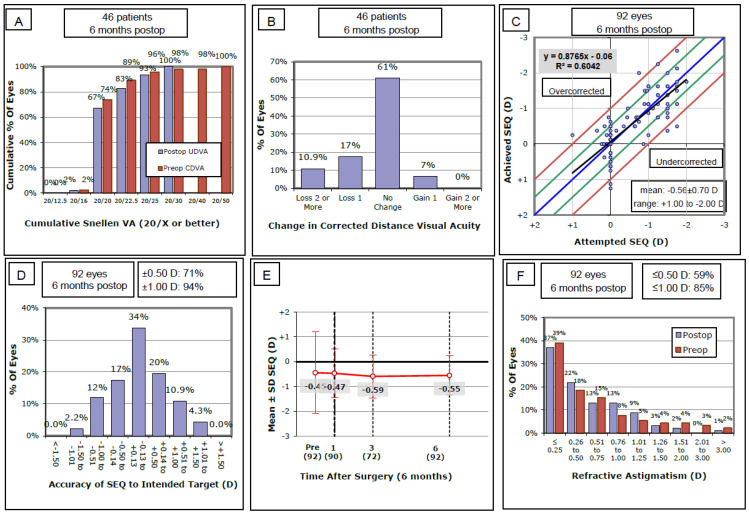
Standard refractive surgery outcome analysis for all 92 eyes (46 patients). (**A**) The cumulative Snellen uncorrected distance VA (UDVA) showing postoperative efficacy. (**B**) A histogram demonstrating the change in corrected distance visual acuity (CDVA) from pre to postoperative measurements. (**C**) A scatter graph for the attempted and achieved spherical equivalent refraction (SEQ). (**D**) A histogram showing the accuracy of the intended spherical equivalent refraction. (**E**) The stability of the spherical equivalent across postoperative follow-up intervals (1, 3, and 6 months). (**F**) A histogram showing the astigmatism postoperatively among all eyes. Abbreviations: UDVA, uncorrected distance visual acuity; CDVA, corrected distance visual acuity; SEQ, spherical equivalent; D, diopters.

A total of 46 patients participated in this study (mean age 47.5 yrs ± 4.7) (92 eyes in total) with presbyopic myopia (31 eyes) (34%), hyperopia (15 eyes) (16%), and emmetropia (46 eyes) (50%). Twenty-eight (61%) of the sample were female. Table 1 shows the demographic data for this sample. Table 2 shows the spherical equivalent baseline and postoperative data. Figure 1 shows the standard graphs for refractive surgery outcomes for this study.

In this study, the mean attempted spherical equivalent for all eyes was −0.56 ± 0.70 (95% CI, −0.70 to −0.41; range: −2.00 D to +1.00 D), while the mean achieved spherical equivalent was −0.55 ± 0.79 (95% CI, −0.71 to −0.38; range: −2.65 D to +1.25 D) (Figure 1C). For distance eyes, the mean attempted spherical equivalent was 0.023 ± 0.27 (95% CI, −0.05 to 0.10; range: −1.50 D to +1.00 D), while the mean achieved spherical equivalent was 0.048 ± 0.43 (95% CI, −0.08 to 0.16; range: −0.75 D to + 1.25 D). For near eyes, the mean attempted spherical equivalent for all eyes was −1.14 ± 0.47 (95% CI, −1.28 to −1.00; range: −2.00 D to 0 D), while the mean achieved spherical equivalent was −1.15 ± 0.58 (95% CI, −1.31 to −0.98; range: −2.62 D to 0 D).

### 3.1. Accuracy

In this study, 71% of distance eyes were within ±0.50 D, and 94% were within ±1.00 D (Figure 1D). Three percent of eyes were overcorrected, and 4% were undercorrected.

### 3.2. Efficacy

The mean logMAR for the UDVA postoperatively and during follow-up at one month, three months, and six months was 0.016, 0.037, and 0.028 (20/20 to 20/22 range), respectively. Postoperatively, 81% of patients achieved 20/22.5 vision or better, 94% achieved 20/25 vision or better, and 100% achieved 20/30 vision or better. The mean difference in spherical equivalent refraction among patients’ eyes postoperatively was 1.22 ± 0.7 D (95% CI, 1.02 to 1.42; range: −2:65 D to +1.25 D). For reading and near work, 100% of patients reached J2 print postoperatively. After treatment, 97% of the patients achieved 20/25 or better at distance and J2 or better at near.

### 3.3. Safety

Figure 1B illustrates the changes in corrected distance visual acuity. Minimal postoperative complications were observed in this sample. Only 5 of 92 eyes (5.4%) suffered from insignificant post-LASIK epithelial ingrowth. Of these, four eyes were emmetropic, and one eye was myopic. Furthermore, only 5 eyes (5.4%) required re-treatment. Re-treatment was performed as an enhancement for residual refractive error, and no cases required reversal of the blended vision protocol due to intolerance.

### 3.4. Stability

The stability of the spherical equivalent refraction over six months of follow-up is presented in Figure 1E. Only three eyes (3%) changed by more than 1.00 D between the 3 and 6 months. The average change in spherical equivalent for the distance (dominant eye) refraction was 0.07 ± 0.64 D and 0.13 ± 0.69 D for near, while for both eyes, it was 0.08 ± 0.88 D.

### 3.5. Cylinder

The spherical group with eyes with less than 0.50 D cylinder had a mean preoperative cylindrical power of −0.4 ± 0.38 (95% CI, −0.51 to −0.29) (33 eyes). After treatment, their mean cylindrical power was −0.55 ± 0.48. Figure 1F shows the amount of astigmatism among patients.

### 3.6. Change in Keratometry

The mean average keratometry postoperatively for all patients was 42.4 ± 2.1 D (95% CI, 41.70 to 43.00; range: 37.2 to 46.9 D). The average keratometry postoperatively for myopes was 41.4 ± 1.7 D, 44.1 ± 1.33 D for hyperopes, and 42.8 ± 1.7 D for emmetropes. Linear regression analysis showed that for every diopter of correction, there was a decrease of 0.75 D (R^2^: 0.77, *p* < 0.001) for myopes and an increase of 0.92 D (R^2^: 0.44, *p* < 0.01) and 1.1 D (R^2^: 0.76, *p* < 0.001) for hyperopes and emmetropes, respectively.

### 3.7. Spherical Aberration

**Figure 2:**
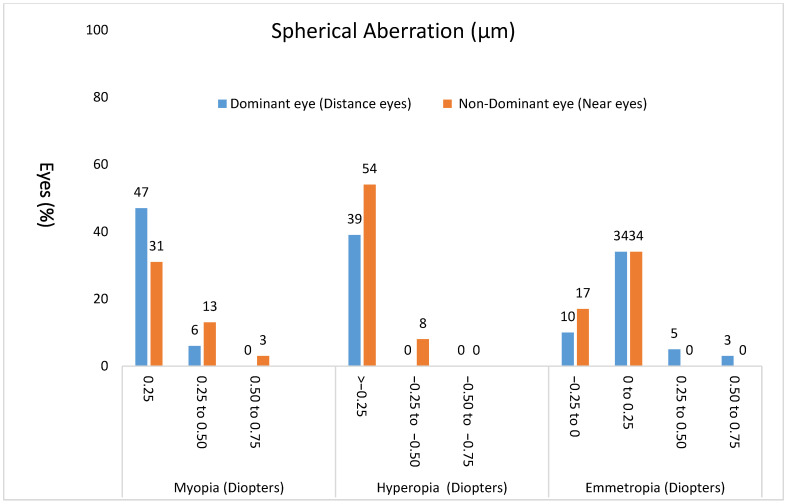
The distribution of the spherical aberration postoperative for myopes, hyperopes, and emmetropes.

The mean spherical aberration (6 mm analysis zone, OSA notation) preoperatively for all eyes was 0.12 ± 0.10 μm (95% CI, 0.09 to 0.14). Postoperatively, the mean spherical aberration for all eyes was 0.06 ± 0.15 μm (95% CI, 0.03 to 0.08). The mean intended spherical aberration was −0.23 ± 0.18 μm. Figure 2 shows the distribution of postoperative spherical aberration in myopes, hyperopes, and emmetropes.

### 3.8. Satisfaction

Patient satisfaction was observed in 42 patients out of the total 46 patients (92% of the cohort), showing that only 4 patients were unsatisfied with the results, 3 patients were unsatisfied with the near VA, and 1 patient was not satisfied with the overall results.

## 4. Discussion

This study demonstrated that the PRESBYOND (LBV) surgery is safe, effective, and provides stable outcomes for patients with emmetropia, hyperopia, and myopia after six months of follow-up with minimal surgical complications. We have also shown that this surgical protocol was associated with a strong level of patient satisfaction among those who were interested in not wearing any optical correction, either contact lenses or glasses. Our findings are consistent with previously published studies evaluating PRESBYOND Laser Blended Vision across different populations. Russo et al. [15] reported that approximately 100% of eyes were within ±1.00 D of the intended correction, compared to 94% in our cohort. Similarly, Reinstein et al. [11,16] demonstrated high levels of binocular visual performance and patient satisfaction following PRESBYOND treatment, supporting the effectiveness of this protocol across their populations.

Our results interestingly showed that 94% of patients reached 20/25 vision or better postoperatively when compared with 96% of patients reaching the same visual acuity under the same binocular conditions preoperatively, even though patients were anisometropic postoperatively. Others reported similar findings, indicating that although the non-dominant eye was corrected for near, it still participates positively in distance vision through neural summation [13,14,15,16,17]. Additionally, 100% of patients achieved J2 print for reading. These findings for both distances indicate that patients’ eyes produce a large intended extended depth of focus designed by the PRESBYOND LBV protocol and produce a beneficial blend zone between the patient’s eyes to be used for distance and near. Other reports showed that using this protocol had not compromised other visual functions, such as contrast sensitivity, stereoacuity, and reading speed [9,15]. However, these parameters were not assessed in our study and therefore cannot be directly evaluated in our cohort.

The surgical efficacy demonstrated good predictability, and the precision of the achieved spherical equivalents for distance eyes in relation to the intended spherical equivalent was 71% within ±0.50 D (R^2^ = 0.82, *p* < 0.001) and 94% (R^2^ = 0.7, *p* < 0.001) within ±1.00 D. This means that 82% of the predictability in the achieved refraction within ±0.50 D can be explained by the regression equation and 70% within ±1.00 D. Others have shown a better prediction rate in their cohort [15]. This could be attributed to the improvement they made in their nomogram, which resulted in better accuracy. In our study, we have used the setting recommended by the manufacturer.

Our study’s surgical output and safety profile demonstrated a strong success and satisfaction rate among patients, despite treating patients with various refractive errors, including emmetropes, hyperopes, and myopes. One attribute of this is the ability to introduce both positive and negative spherical aberration to the patient’s intended extended depth of focus [13,18]. Ninety-two percent of our patients were satisfied with the results. Similar satisfaction rates were observed across different populations and ethnicities [9,15,19]. Although anisometropia is reversible, none of the unsatisfied patients (8%) requested re-treatment or reversal of the anisometropia. The patient adaptation process was evaluated through the follow-up sessions. This was monitored through the distance and near VA, as well as the patient’s satisfaction rate. The patients’ average distance LogMAR VA at 1, 3, and 6 months was 0.016, 0.037, and 0.028 (20/20 to 20/22 range), respectively. This indicated that a small change in VA was observed over time, with a slight reduction in acuity at 3 months and a mild improvement by 6 months. Even though this change in VA is minimal and remains within the range of good functional vision, it likely reflects the intended induction of anisometropia and the inherent trade-off of the protocol between slightly reduced distance VA and an improved near VA. For near VA, 100% of our patients reached J2 print. This was observed 1 month postoperatively and remained unchanged at the 6-month follow-up session. Brar et al. [9] reported comparable outcomes in reading performance and functional vision, with patients achieving high levels of near visual acuity and maintaining good distance vision. It should be noted that social reading activities for everyday use, which include classified advertisements, stock quotes, regular newspapers, a phone book, articles from journals and magazines, and packets of sweetener, require a maximum near VA of J5 print [19]. PRESBYOND LBV protocol in our study and others has been shown to provide better near visual acuity than what is required in social reading activities for everyday use. It is unsurprising that different populations, under the care of various surgeons, achieve this level of patient satisfaction when using this protocol, as both distance and near visual acuities are usable, and patients are independent of using any refractive aids.

The patient satisfaction in our study was assessed using a self-developed binary (Yes/No) survey. While this approach might provide a general assumption about patient satisfaction, it does not capture quantitative or domain-specific insights into visual quality or performance. Therefore, the satisfaction rate in this study should be interpreted with caution. It would be ideal, however, to evaluate the patient’s quality of vision postoperatively using structured questionnaires such as the Quality of Life Impact of Refractive Correction (QIRC) [20] or the Visual Function Index (VF-14) [21]. The use of validated, multidimensional questionnaires would enable assessment across multiple domains, providing a deeper understanding of the patient’s needs and advancing the development of the protocol.

Different studies have shown that older adults can suffer from various binocular vision disorders, and these disorders increase with age and can result in reduced stereoacuity, diplopia, and asthenopia [22,23,24]. Abnormal binocular vision is associated with reduced balance, fractures, musculoskeletal injuries, visuomotor tasks, and falls among older adults [22,23,25]. The ability of a person to integrate and synchronize various visual functions, including binocular vision, can effectively improve patients’ reading abilities and visual function and reduce the associated risks. Therefore, since PRESBYOND LBV patients are anisometropic, the results in this study and other studies suggest that this protocol offers an effective compromise between near and distance visual demands, minimizing the stereopsis loss typically observed with conventional monovision. Nevertheless, further work is warranted to evaluate the status of binocular vision, visual fixation, binocular fusion, visual tracking, hand–eye coordination, convergence, and convergence efficiency to help improve the prediction outcome of the PRESBYOND LBV protocol for this cohort.

Our study had several limitations. First, the sample size of 46 patients, while valuable, is relatively small and may limit the generalizability of the findings to the broader Saudi population. Additionally, the follow-up period of six months, though sufficient to assess short-term outcomes, may not capture long-term stability, visual quality, or late-onset complications. Furthermore, the study did not include other direct functional assessments such as contrast sensitivity or stereoacuity. Although these parameters are critical for evaluating visual quality, they were beyond the scope of this retrospective design. Future prospective studies should include these measures to provide a more comprehensive understanding of postoperative visual performance. Furthermore, advanced analyses such as vector analysis of refractive error, higher-order aberrations, and corneal shape assessment were not included. These parameters were beyond the scope of the current study. Future studies incorporating these objective measurements and subgroup comparisons are warranted to further optimize patient selection and surgical outcomes. While the study design and outcome measures are consistent with previously published refractive surgery reports, this study contributes region-specific clinical data from a Saudi cohort, where evidence remains limited. Further subgroup analyses and comparative studies may provide additional insights into refractive subgroup differences and enhance the generalizability and clinical applicability of the findings.

## 5. Conclusions

To conclude, the PRESBYOND LBV protocol demonstrated stable, well-tolerated, effective, and acceptable results in hyperopic and myopic patients with presbyopia over a 6-month observation period among the Saudi population. The safety and efficacy of the procedure were favorable and strong. The patients’ satisfaction rate was 92%, with only 8% of patients reporting dissatisfaction. These strong outcomes, along with the simplicity and minimal invasiveness, make this protocol a viable solution for surgeons and patients to consider for presbyopia. However, further studies incorporating additional functional visual outcomes and longer follow-up periods are warranted to provide a more comprehensive evaluation of postoperative visual performance.

## Figures and Tables

**Table 1:** Shows demographic and preliminary data for different parameters.

Parameter	Demographic Data	
Age	47.5 yrs ± 4.7	
No of eyes	92	
Sex	61% Females	
Dominant eye	37% OD	63% OS
Ablation depth (μm)	40.50 ± 22	
Optical zone (mm)	6.48 ± 0.08	
Residual stromal thickness (RST) (μm)	381 ± 37	
Central corneal thickness (CCT) (μm)	533 ± 29	
Parameter	Preoperative	Postoperative (6 months)
Binocular Visual acuity (LogMAR)	0.004 (20/20) ± 0.02	0.028 (20/22) ± 0.05
Keratometry (D)	42.54 ± 1.80	42.59 ± 2.20
Spherical aberration Z40	0.12 ± 0.10	0.06 ± 0.15
Coma	0.17 ± 0.10	0.24 ± 0.20

**Table 2:** Spherical equivalent at baseline and postoperative.

Outcome	Dominant Eye (Distance)	Non-Dominant Eye (Near)	*p*
Mean ± SD (Range, D)			
Preoperative	−0.42 ± 1.53 (−3.75 to +2.87)	−0.4 ± 1.76 (−4.00 to +3.12)	0.90
Postoperative 1 month	0.12 ± 0.79 (−1.25 to +4.5)	−1.05 ± 0.74 (−2.62 to +1.62)	<0.01
Postoperative 3 months	0.01 ± 0.64 (−1.75 to +1.75)	−1.20 ± 0.59 (−2.62 to 0)	<0.01
Postoperative 6 months	0.048 ± 0.43 (−0.75 to +1.25)	−1.15 ± 0.58 (−2.62.00 to 0)	<0.01

*p* values represent paired comparisons between dominant and non-dominant eyes at the patient level.

## Data Availability

The datasets generated in this study are available from the corresponding author upon request.
